# Identification and prognostic value of DLGAP5 in endometrial cancer

**DOI:** 10.7717/peerj.10433

**Published:** 2020-11-27

**Authors:** Ruoyi Zheng, Zhengzheng Shi, Wenzhi Li, Jianqin Yu, Yuli Wang, Qing Zhou

**Affiliations:** 1 Department of Gynecology, the First Affiliated Hospital of Wenzhou Medical University, Wenzhou, Zhejiang, China; 2 Obstetrics and Gynecology Hospital, Fudan University, Shanghai, China

**Keywords:** Endometrial carcinoma, DLGAP5, Prognosis, Kaplan–Meier plotter, TCGA-UCEC, The Human Protein Atlas

## Abstract

**Background:**

Endometrial cancer poses a serious threat to women’s health worldwide, and its pathogenesis, although actively explored, is not fully understood. DLGAP5 is a recently identified cell cycle-regulation gene not reported in endometrial cancer. This study was aiming to analyze the role of DLGAP5 in tumorigenesis and development and to investigate its prognostic significance of patients with endometrial cancer.

**Methodology:**

Microarray datasets (GSE17025, GSE39099 and GSE63678) from the GEO database were used for comparative analysis, and their intersection was obtained by applying the Venn diagram, and DLGAP5 was selected as the target gene. Next, transcriptome data (*n* = 578) was downloaded from TCGA-UCEC to analyze the mRNA expression profile of DLGAP5. Then, immunohistochemical data provided by HPA were used to identify the different protein expression levels of DLGAP5 in tumor tissues and normal tissues. Subsequently, the prognostic meaning of DLGAP5 in patients with endometrial cancer was explored based on survival data from TCGA-UCEC (*n* = 541). Finally, the reliability of DLGAP5 expression was verified by RT-qPCR.

**Results:**

Transcriptome data from TCGA-UCEC, immunohistochemical data from HPA, and RT-qPCR results from clinical samples were used for triple validation to confirm that the expression of DLGAP5 in endometrial cancer tissues was significantly higher than that in normal endometrial tissues. Kaplan–Meier survival analysis announced that the expression level of DLGAP5 was negatively correlated with the overall survival of patients with endometrial cancer.

**Conclusions:**

DLGAP5 is a potential oncogene with cell cycle regulation, and its overexpression can predict the poor prognosis of patients with endometrial cancer. As a candidate target for the diagnosis and treatment of endometrial cancer, it is worthwhile to make further study to reveal the carcinogenicity of DLGAP5 and the mechanism of its resistance of organisms.

## Introduction

Since the end of the 20th century, the incidence of endometrial cancer has continued to rise globally, but the survival rate has not improved  (Bray et al., 2018). The American Cancer Society released a report in early 2019, predicting that the number of new cases of endometrial cancer in 2019 would be 1.24 times that of all other female reproductive malignancies  (Siegel, Miller & Jemal, 2019). Under the severe situation, the field of bioinformatics technology has developed rapidly in recent years, and many online database platforms have sprung up, which are often used to explore new biomarkers, as targets for diagnosis and treatment, and even to evaluate prognosis.

The essential cause of tumor formation is attributed to the uncontrollable cell division, which leads to infinite proliferation. Therefore, regulation of cell cycle becomes a crossroad for tumorigenesis or tumor suppression. Mitosis, as a specific process of cell cycle, involves many complicated steps. According to the morphology of chromosomes and DNA synthesis in cells, mitosis is divided into G1, S, G2, and M phases. Many cycle-related proteins have been identified, containing the cyclin family, cyclin-dependent kinase (CDK) family, maturation promoting factor (MPF) and P53  (Strohmaier et al., 2001; Navarro & Nurse, 2012; Gavet & Pines, 2010; Liao et al., 2017).

Five members of the disc large-associated protein (DLGAP) family, named DLGAP1, 2, 3, 4, and 5, are directly associated with a variety of psychological and neurological disorders but have been less reported in cancer  (Rasmussen, Rasmussen & Silahtaroglu, 2017). Among them, DLGAP5 is the member most closely related to carcinoma, but there are very few studies in the field of endometrial cancer. The expression of DLGAP5 is periodic and has the function of promoting microtubule polymerization and bipolar spindle formation, which is necessary to maintain the growth and stability of microtubules in the spindle and facilitate the cell mitosis process pass the G2/M checkpoint (Wong & Fang, 2006; Koffa et al., 2006).

For this research, we verified the differential expression of DLGAP5 (ENSG00000126787) in endometrial cancer and normal endometrial tissue with the help of bioinformatics tools, further analyzed the possible mechanism of its dysregulation, and assessed the significance of its prognostic.

## Materials and Methods

### Bioinformatic data mining in Gene Expression Omnibus (GEO)

Three normalized microarrays, GSE17025  (Day et al., 2011), GSE39099 and GSE63678 (Pappa et al., 2015), were obtained from the GEO database  (Barrett et al., 2013). The GSE17025 dataset contained 91 stage I EC samples, which were composed of 79 endometrioid and 12 serous, and 12 normal endometrium samples from postmenopausal women. The GSE39099 dataset contained 40 endometrial samples divided into four groups, which consisted of normal endometrium (NEM, 10), atypical endometrial hyperplasia (AEH,10), early-staged EC (stages I and II, 10) and advanced-staged EC (stages III and IV, 10). The GSE63678 dataset contained 35 gynecological cancers samples, five of which were NEM and seven of which were EC. The GSE17025 and GSE39099 datasets were analyzed on the GPL570 platform (Affymetrix Human Genome U133 Plus 2.0 Array; Affymetrix, Inc., Santa Clara, CA, USA), while the GSE 39099 had not published yet. The GSE63678 dataset was analyzed on the GPL571 platform (Affymetrix Human Genome U133A 2.0 Array; Affymetrix, Inc., Santa Clara, CA, USA). The interactive online tool GEO2R did the standardization and analysis of the raw data.

### Immunohistochemistry (IHC) analysis in the Human Protein Atlas

The Human Protein Atlas database (HPA)  (Uhlen et al., 2017) contained six separate parts: cells, tissues, organs, etc., which mapped human proteins from a diverse perspective. This database provided information on the expression and localization of human proteins in 44 different tissue types and various organs. Differential expression of DLGAP5 protein level between normal endometrial tissues and EC tissues was evaluated online with immunohistochemical staining data offered by HPA.

### Analysis of web bioinformatics tools based on the data of The Cancer Genome Atlas -Uterine Corpus Endometrial Carcinoma (TCGA-UCEC)

HPA divided the patients into two groups after determining the best expression cut-off value of the Fragments Per Kilobase of transcript per Million fragments mapped (FPKM) based on TCGA-UCEC. Interactive survival scatter plot and Kaplan–Meier curve were drawn based on prognosis (survival) and DLGAP5 expression (FPKM). Kaplan–meier Plotter (K-M plotter)  (Gyorffy, Lánczky & Szállási, 2012), an online tool focused on survival analysis, plotted the survival curves of 37 DEGs based on the best cut-off in TCGA-UCEC data.

### Real-time quantitative polymerase chain reaction (RT-qPCR) validation

A total of 16 pairs of clinical samples obtained from patients who underwent a hysterectomy in the First Affiliated Hospital of Wenzhou Medical University between 2016 and 2019. The data in this study consisted medical records or specimens collected with informed consent from previous research. None of the patients had received chemotherapy or radiotherapy before surgery, had no history of other malignant tumors, and had signed informed consent forms before surgery. The study was approved by the Ethics Committee in Clinical Research of the First Affiliated Hospital of Wenzhou Medical University (Ethics committee issuing number: 2020–028). And The First Affiliated Hospital of Wenzhou Medical University waived the need to obtain additional consent from the human subjects for this study.

RT-qPCR detected differential expression of DLGAP5 molecular level between normal endometrial tissues and EC tissues. Total RNA was extracted with Trizol reagent (Invitrogen, Carlsbad, CA, USA). The messenger RNA was reverse transcribed into cDNA using the Prime Script RT reagent Kit (TaKaRa, Shiga Prefecture, Kusatsu, Japan). Real-time fluorescent quantitative PCR reactions were performed using the SYBR Premix Ex Taq II kit (TaKaRa, Shiga Prefecture, Kusatsu, Japan). Primer sequences were as follows: the DLGAP5 primers, forward: 5′- GTTGTGCAGCCTGTAATGCC-3′, reverse: 5′- TAGCAGCTCTTGTGACTGGC-3′; and ACTB primers, forward, 5′- CATCCCCCAAAGTTCACAAT-3′, reverse: 5′- AGTGGGGTGGCTTTTAGGAT-3′. The RT-qPCR system was QuantStudio 3 (Applied Biosystems, Foster City, CA, USA), and the data were processed using the QuantStudio 3 design & analysis software 1.4 (Applied Biosystems) based on comparative cycle threshold (2^−ΔΔCt^) values  (Livak & Schmittgen, 2001).

### Statistical analysis

Based on the FPKM value of DLGAP5, HPA was used to analyze the survival of patients with endometrial cancer in TCGA-UCEC by the Kaplan–Meier method with the log-rank test. Other data were carried out by SPSS 20.0 (IBM, Chicago, IL). The significance of HTseq-TPM expression of DLGAP5 in endometrial cancer tissues and normal endometrial tissues from TCGA-UCEC was determined by the Independent Samples *t*-test (Mann–Whitney test was used if the data did not follow the normal distribution)  (Le et al., 2019b; Le et al., 2019a). The expression of DLGAP5 in clinical endometrial cancer tissues and adjacent normal tissues was analyzed by the Paired Samples *t*-test (Wilcoxon test was used if the data did not follow the normal distribution). Data for at least three independent experiments were recorded as the mean standard deviation (mean ± SD). Significance was defined as *P* < 0.05.

## Results

### DLPAG5 is an up-regulated Differentially Expressed Gene (DEG) in endometrial cancer

**Figure 1:**
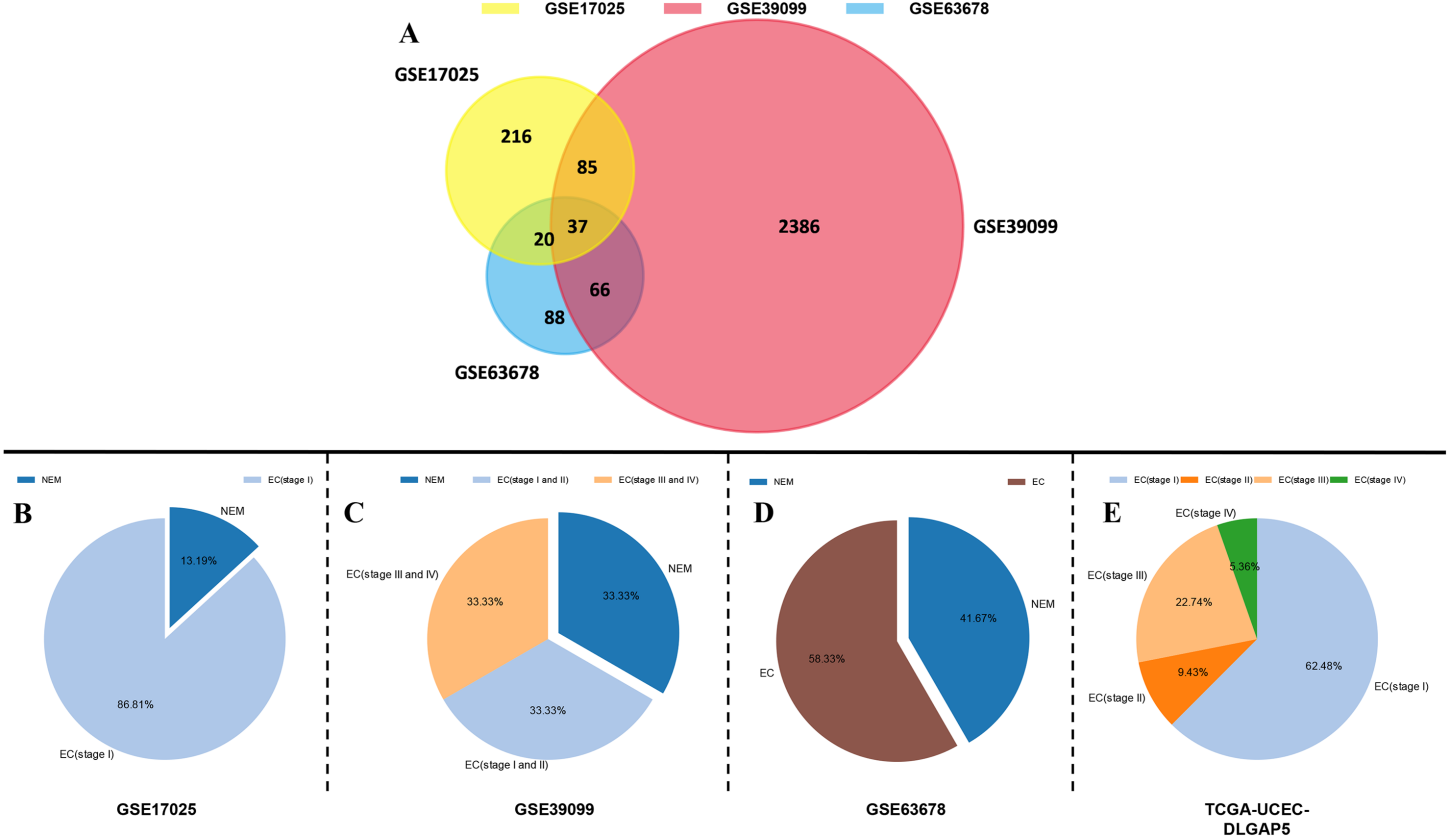
DEGs of EC were identified from the three datasets. (A) The central overlap area represented their consistent set. *P* < 0.05 and |log(fold-change)| > 2 intercepted statistically significant DEGs. (B–E) The overall tumor staging distribution of GSE17025, GSE39099, GSE63678 and TCGA-UCEC-DLGAP5.

**Figure 2:**
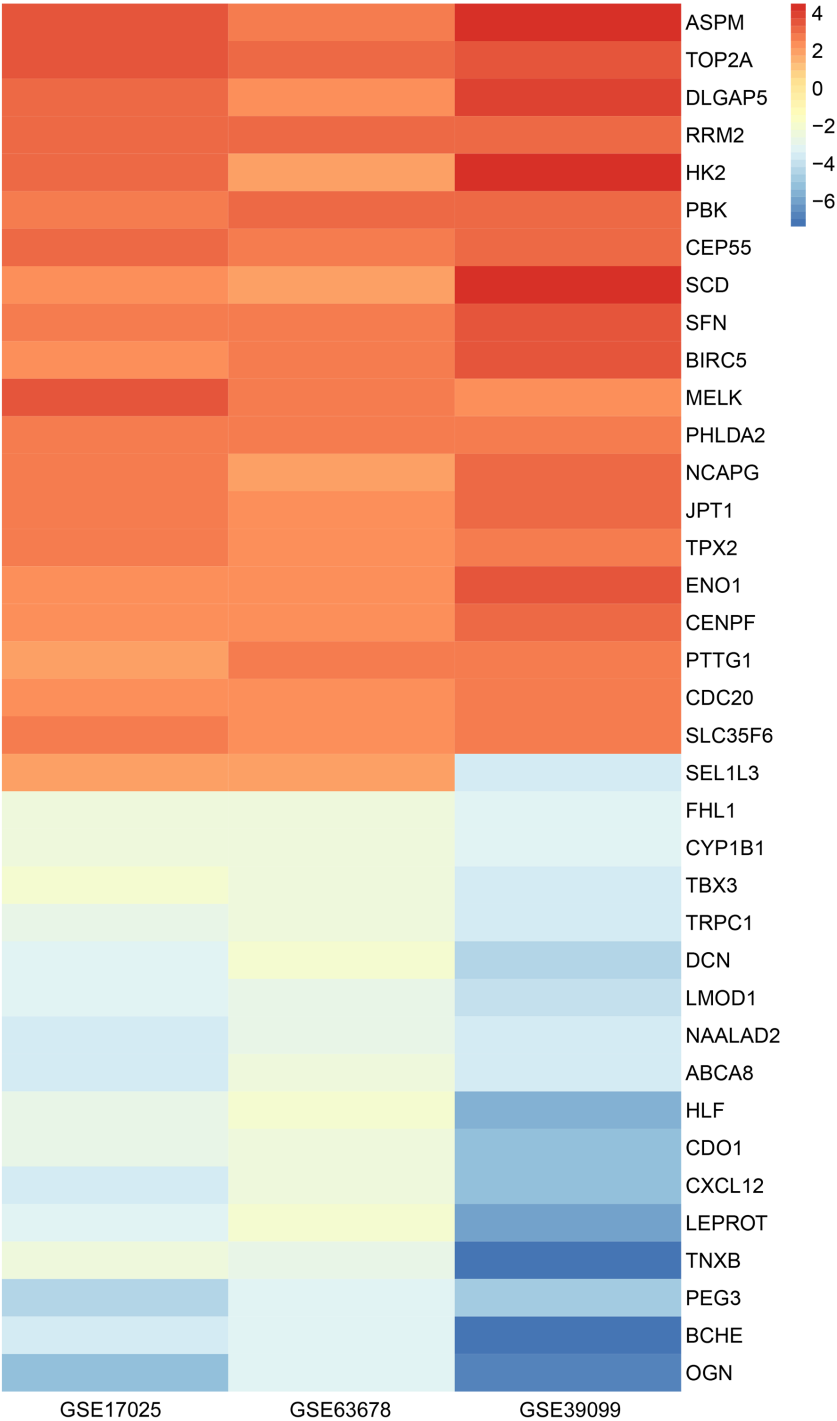
Heatmap of 37 DEGs in GSE17025, GSE39099, and GSE63678. Representative immunohistochemical images of DLGAP5 protein were presented. (A) Negative staining in normal endometrial tissue. (B) low staining in normal endometrial tissue. (C) Negative staining in endometrial cancer tissue. (D) Low staining in endometrial carcinoma tissues. (E) Moderate staining of endometrial carcinoma tissue. The images above were obtained from HPA with the patient ID as the unique identification code.

Through the mining of the GEO database by GEO2R, we extracted DEGs of endometrial cancer from the three datasets of GSE17025, GSE39099, and GSE63678. With *P* < 0.05 and —log(fold-change)— >  2 as the screening criteria, 358 DEGs were identified from GSE17025, 211 from GSE39099 and 2574 from GSE63678. Using the Venn diagram to take the intersection of the three, a total of 37 overlapping values could be obtained, and the overall tumor staging distribution in all the original data of the study was demonstrated (Fig. 1). Meanwhile, the cluster heatmap presented the expression profiles of 37 DEGs in three datasets. (Fig. 2, Table 1). DLGAP5, which had rarely studied in EC, was selected as the study object (Table 2).

### Histopathological expression of DLPAG5 in the endometrium

**Figure 3:**
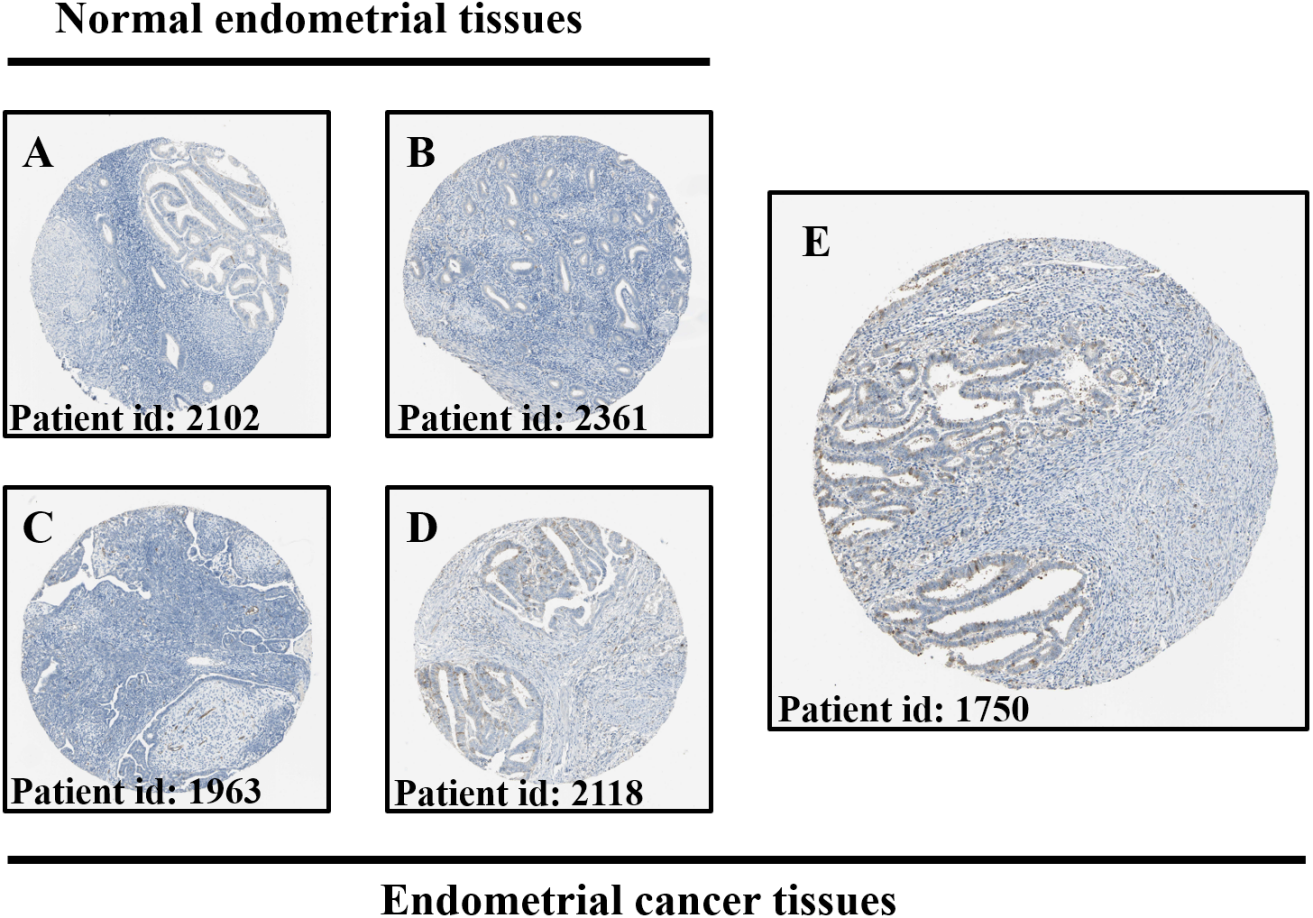
Comparison of protein expression of DLGAP5 in normal endometrial tissues and endometrial cancer tissues. Representative immunohistochemical images of DLGAP5 protein were presented. (A) Negative staining in normal endometrial tissue. (B) Low staining in normal endometrial tissue. (C) Negative staining in endometrial cancer tissue. (D) Low staining in endometrial carcinoma tissues. (E) Moderate staining of endometrial carcinoma tissue. The images above were obtained from HPA with the patient ID as the unique identification code.

To clarify the clinical relevance of DLGAP5, we used the Human Protein Atlas database to analyze the expression of DLGAP5 in normal endometrial tissues and endometrial cancer tissues. In the only two normal endometrial tissues, DLGAP5 was low stained in the endometrial stroma cells in one case and undetected in the other. By comparison, among the 11 endometrial cancer tissues examined, moderate and negative DLGAP5 staining were in a clear minority (18%), and the majority was low-stained (64%) (Fig. 3).

### Expression and prognostic significance of DLGAP5

**Figure 4:**
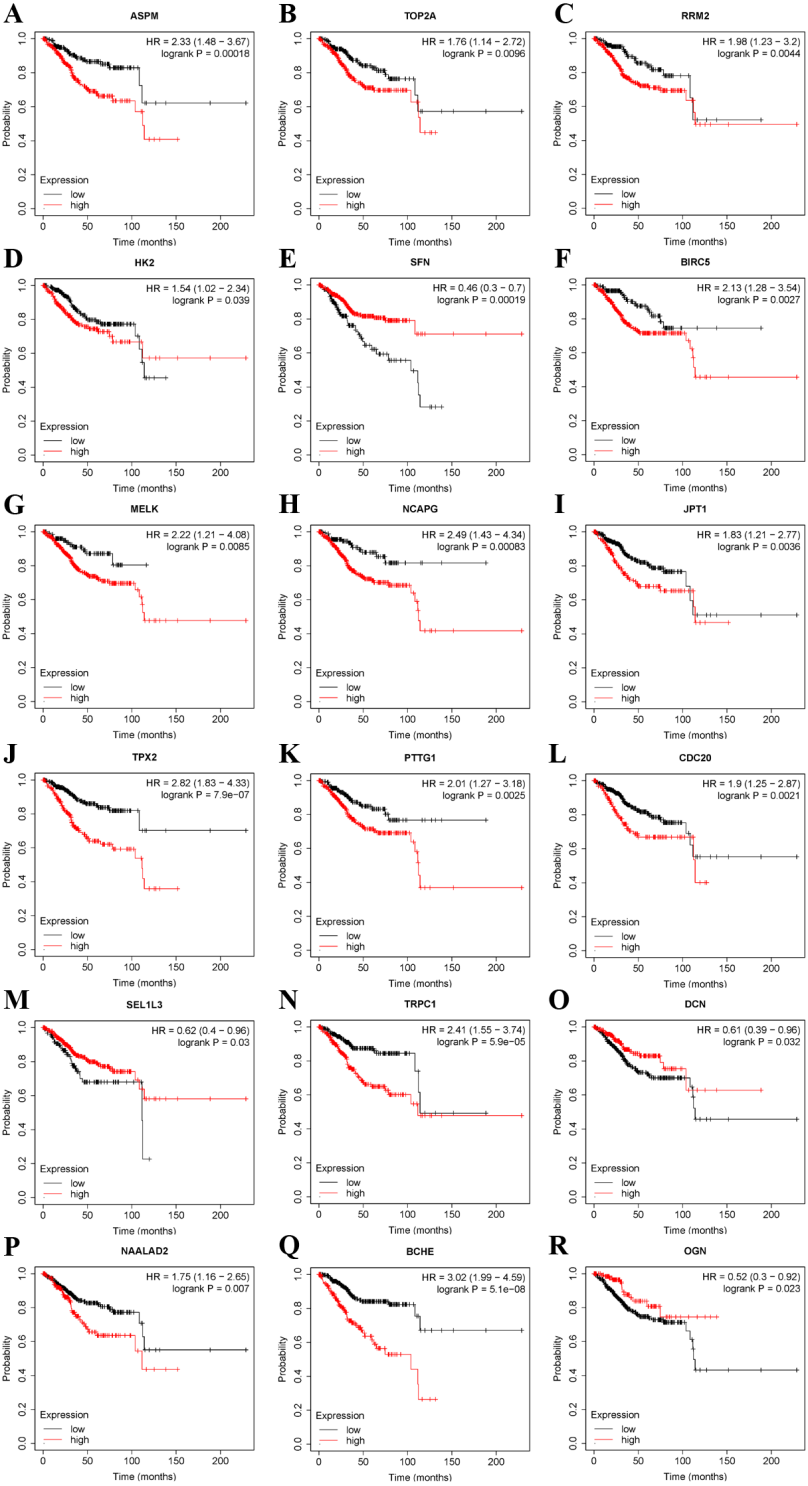
Kaplan–Meier survival plots of 18 DEGs. Red line represents higher expression of the gene, black one lower expression. *X* axis means survival time (month). *Y* axis means survival probability. Hazard ratio and confidence interval are calculated. Logrank *P* < 0.05 means that the gene is significantly associated with the prognosis of endometrial cancer. (A) ASPM. (B) TOP2A. (C) RRM2. (D) HK2. (E) SFN. (F) BIRC5. (G) MELK. (H) NCAPG. (I) JPT1. (J) TPX2. (K) PTTG1. (L) CDC20. (M) SEL1L3. (N) TRPC1. (O) DCN. (P) NAALAD2. (Q) BCHE. (R) OGN.

Most of the 37 DEGs identified in this study are novel and not well studied. In particular, LEPROT, HLF, ABCA8, LMOD1, TRPC1, FHL1, SLC35F6, and SEL1L3 have not been reported in the field of endometrial cancer. Based on TCGA data, the influence of these 37 DEGs on survival probability was carried out by the Kaplan–Meier plotter online tool. Apart from DLGAP5, there were still 18 genes significantly correlated with the prognosis of endometrial cancer (*P* < 0.05) (Fig. 4). The other 18 genes appeared to be associated only with the development of endometrial cancer.

**Figure 5:**
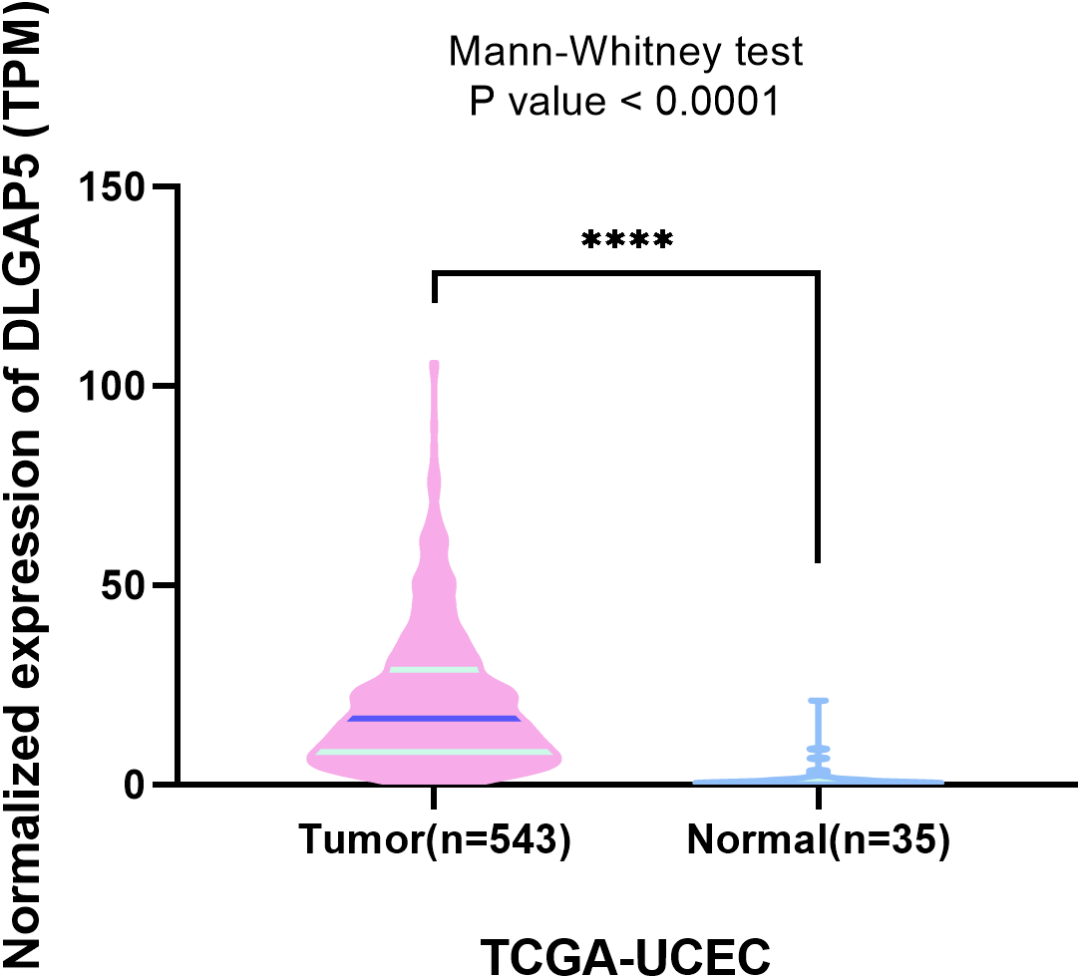
Comparison of HTseq-TPM expression in endometrial cancer tissues (*n* = 543) and normal endometrial tissues (*n* = 35) of DLGAP5. In the violet plot, the blue lines represented the median and the pale green lines represented the quartiles. Man–Whitney test was used, *P* < 0.0001.

The complete HTseq-FPKM data was packaged and downloaded from TCGA-UCEC, and then converted into Transcripts per million reads (TPM) utilizing data normalization. By contrast, we found that the expression of DLGAP5 in endometrial cancer tissues was significantly higher than that in normal endometrial tissues (*P* < 0.0001) (Fig. 5).

**Figure 6:**
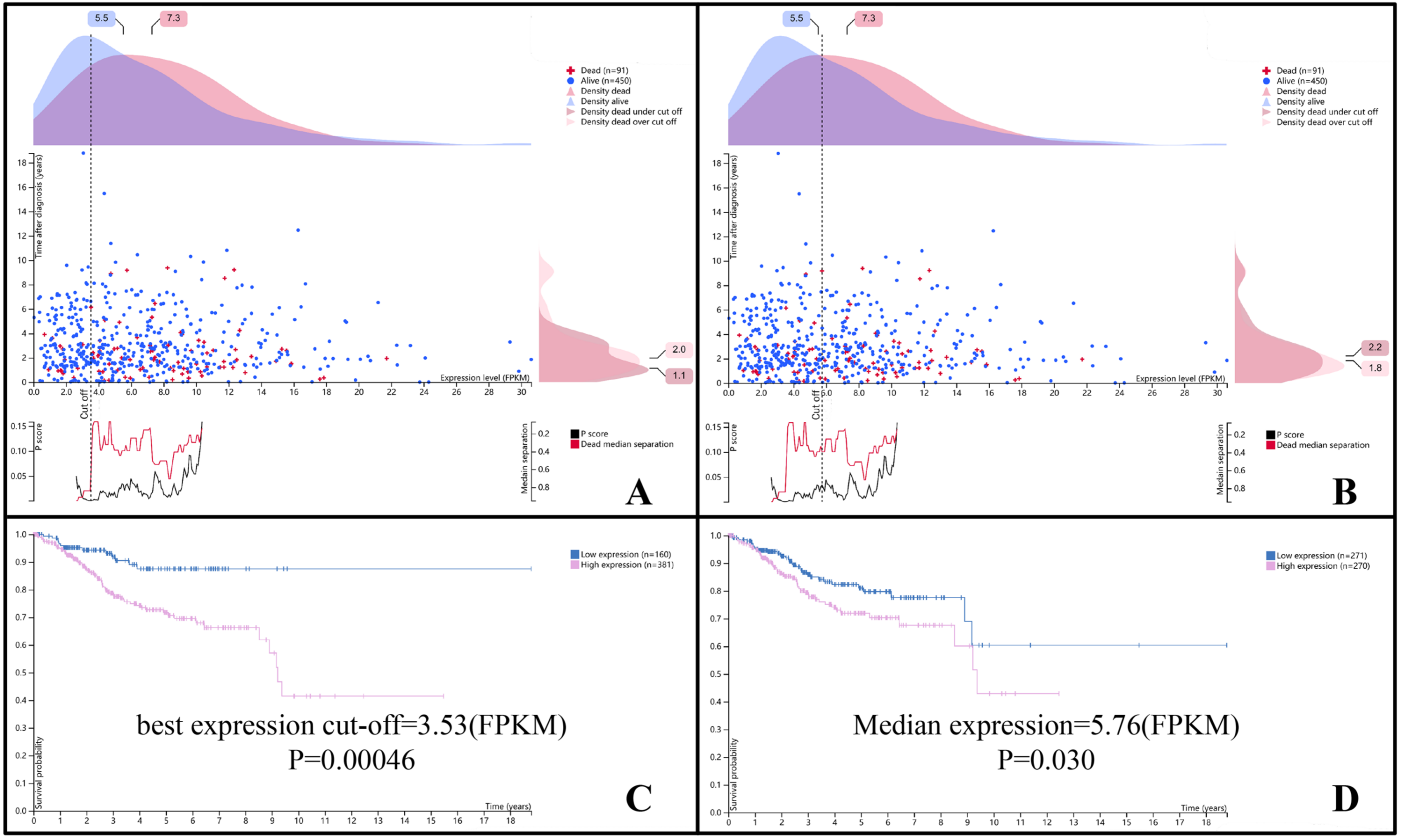
The prognostic value of DLGAP5 expression (FPKM) in OS of endometrial cancer patients in TCGA-UCEC (A) The survival scatter plot with the best expression cut-off (FPKM = 3.53). (B) The survival scatter plot with median expression (FPKM = 5.76). (C) The Kaplan–Meier plot with the best expression cut-off (FPKM = 3.53), *P* = 0.00046. (D) The Kaplan–Meier plot with median expression (FPKM = 5.76), *P* = 0.030. The survival scatter plot, based on data consistent with the Kaplan –Meier plot, showed the clinical status (i.e., dead or alive) of all patients at the end of follow-up.

Based on the clinical data of TCGA-UCEC, HPA extracted the survival data of follow-up patients and examined the relationship between overall survival (OS) and DLGAP5 expression (FPKM). The survival analysis included a total of 450 survivors and 91 deaths. According to the FPKM value of DLGAP5, the best expression cut-off value was selected and divided into two groups, and the interactive scatter plot and Kaplan–Meier plot were presented simultaneously (Fig. 6). When the best cut-off value was 3.53, 160 cases in the low-expression group and 381 cases in the high-expression group were obtained (*P* = 0.00046). When the cut-off value selected as the median FPKM value of 5.76, 271 cases were in the low-expression group, and 270 cases were in the high-expression group (*P* = 0.03).

**Table 1:** Thirty-seven DEGs in EC (21 up-regulated and 16 down-regulated), according to the data consolidation analysis of GSE17025, GSE39099 and GSE63678. DEGs in groups are sorted by the magnitude of the | log(fold-change) | value.

Groups	DEGs
Up-regulated	ASPM, TOP2A, **DLGAP5**, RRM2, HK2, PBK, CEP55, SCD, SFN, BIRC5, MELK, PHLDA2, NCAPG, JPT1, TPX2, ENO1, CENPF, PTTG1, CDC20, SLC35F6, SEL1L3
Down-regulated	OGN, BCHE, PEG3, TNXB, LEPROT, CXCL12, CDO1, HLF, ABCA8, NAALAD2, LMOD1, DCN, TRPC1, TBX3, CYP1B1, FHL1

**Table 2:** The expression of DLGAP5 in GSE17025, GSE39099 and GSE63678.

Dataset	*P*-Value	log(fold-change)
GSE17025	8.01 ×10^−11^	3.14
GSE63678	2.17 ×10^−2^	2.46
GSE39099	1.36 ×10^−2^	4.06

### Validation of DLGAP5 in clinical samples

**Figure 7:**
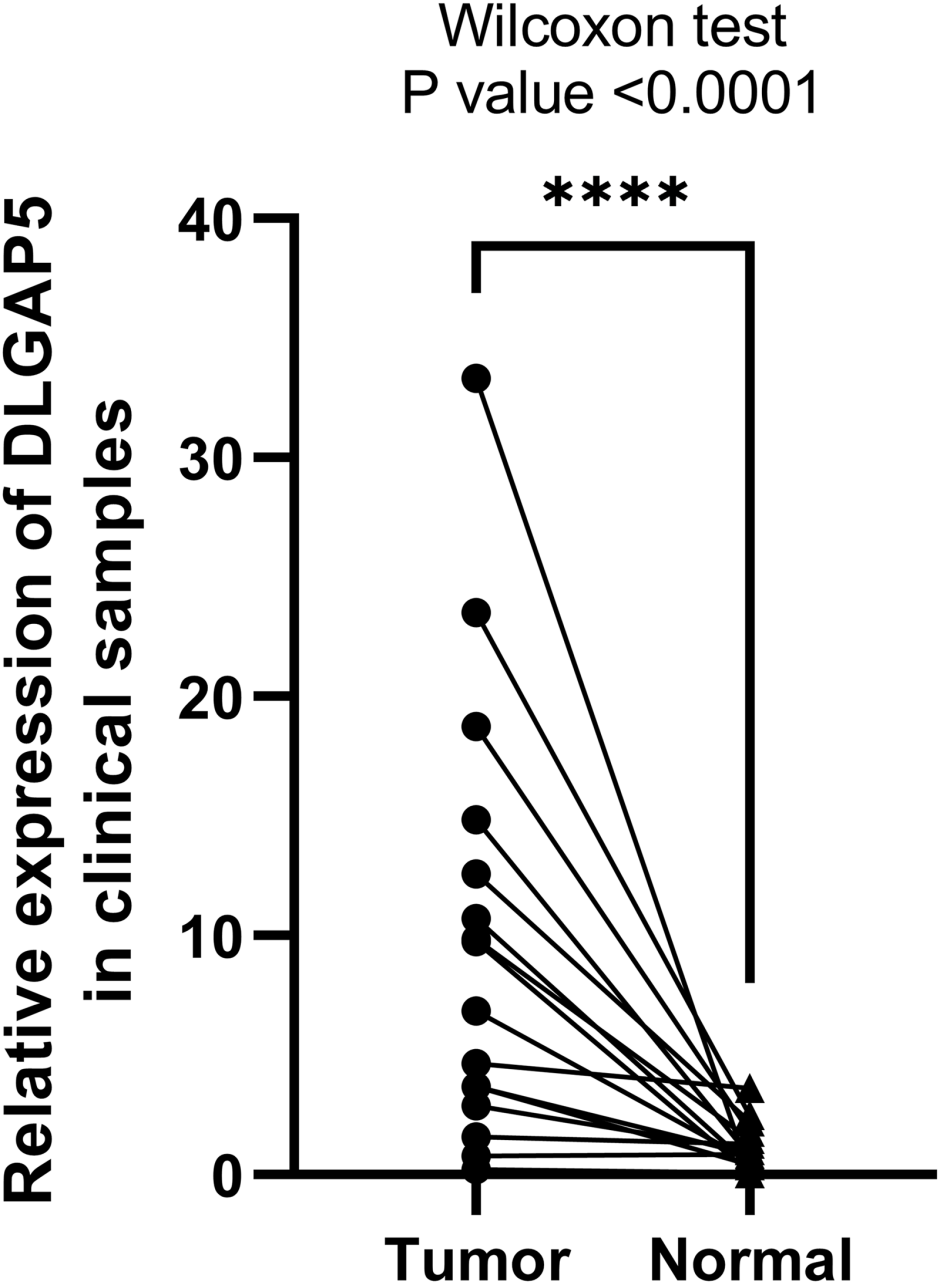
Comparison of the relative expression of DLGAP5 in 16 patients with endometrial cancer and adjacent tissues (*n* = 32). Wilcoxon test was used, *P* < 0.0001.

Back to the clinical samples, taking RT-qPCR to identify the differential expression level of DLGAP5 in tumor and para-cancer tissues of 16 pairs of patients with endometrial cancer. The experimental results are consistent with the conclusions of the bioinformatics analysis (Fig. 7). The expression of DLGAP5 in endometrial cancer tissues was significantly higher than that in adjacent tissues (*P* < 0.001).

## Discussion

DLPAG5, also known as Human hepatoma up-regulated protein (HURP), is overexpressed in a variety of malignant tumors such as hepatocellular carcinoma, non-small cell lung cancer, and ovarian cancer  (Tsou et al., 2003; Tagal et al., 2017; Liu et al., 2019a). DLPAG5 is a component of centromeres that stabilizes microtubules, induces the formation of protein sheets at the end of the microtubules, and ensures the formation of bipolar spindles and the separation of sister chromatids  (Wang et al., 2018; Schneider et al., 2017). As a cell cycle regulating gene involved in mitosis, the expression of DLGAP5 is also cyclical, appearing in the S phase, reaching a peak in G2/M phase and then decreasing in G1 phase, so G2/M phase is also a vital stage for DLGAP5 to play a regulatory role (Wang et al., 2018). DLGAP5 is a substrate of microtubule-related protein Aurora kinase A, and the nuclear localization activity of DLGAP5 is enhanced after phosphorylation. Interestingly, unphosphorylated and phosphorylated DLGAP5 is in dynamic equilibrium, that is, between the cytoplasm and the nucleus  (Chen et al., 2015). Besides, DLGAP5 contains a guanylate-kinase-associated protein (GKAP) domain, which is highly conserved in different species. Simultaneously, this domain also exists in many eukaryotic signaling proteins, which means that DLGAP5 may be a signaling molecule with critical biological functions (Shi et al., 2017).

Although the abnormal expression of DLGAP5 in endometrial cancer has been mentioned in a few literatures, it has not been discussed in depth  (Liu et al., 2019b; Zhang et al., 2020). As a prognostic factor, high expression of DLGAP5 is unfavorable in endometrial cancer. This study found that, in endometrial cancer tissues, both RNA and protein expression levels of DLGAP5 were significantly higher than in normal tissues. The previous research has reported that DLGAP5 is one of the identified crucial mitotic regulators, which interferes with the assembly of the spindle and the separation of sister chromatids (Schneider et al., 2017). So it is foreseeable that the down-regulation of DLGAP5 expression level can block cell cycle in G(2)-M phase, thus inhibiting the proliferation of tumor cells. Also, the anti-cancer effect of silencing DLGAP5 has confirmed in other vitro cell experiments, which may also be related to the fact that DLGAP5 can weaken the ubiquitination and proteasome degradation of p53, or become a direct target gene of the NOTCH3 signaling pathway  (Chen et al., 2012; Kuo et al., 2012). The growth rate of tumor tissues depends to some extent on the activity of mitosis, so the importance of DLGAP5 in tumor generation and development is undeniable. Therefore, we concluded that the upregulation of DLGAP5 was a critical mechanism leading to abnormal proliferation of endometrial cancer cells.

Based on the comparative study of TCGA-UCEC patients’ survival data and clinical samples, we found that high DLGAP5 expression was robustly relevant to the poor OS. In the face of adverse outcomes in patients with endometrial cancer, interventions targeting predisposing factors should probably pay more attention to molecular-level variation. Studies have shown that DLGAP5 may be a potential clinical marker for patients with endometrial cancer and exhibit a variety of tumor-related biological characteristics.

The CpG island methylation level of DLGAP5 promoter can directly regulate the amplification of DLGAP5, achieve epigenetic regulation, and promote the occurrence of liver cancer (Liao et al., 2013). Similarly, we hypothesized that the extent of methylation is also one of the essential mechanisms underlying the dysregulation of DLGAP5 expression in endometrial cancer. DLGAP5 expression is sensitive to hypoxia, which can cause spindle assembly and cytokinesis defects in mammalian cells  (Platani et al., 2015). In prostate cancer cells, DLGAP5 has been proved to be poorly expressed in hypoxic environments and has predictive value for the prognosis of prostate cancer patients  (Espinoza et al., 2016; Gomez et al., 2013). Consequently, taking advantage of the hypoxic-controlled biological properties of DLGAP5, hypoxia-related therapies targeted at tumors may benefit patients with endometrial cancer.

It is noteworthy that DLGAP5 was prominently associated with resistance to chemotherapy drugs and radiotherapy. Under the condition of high expression of DLGAP5, the sensitivity of Hepatocellular carcinoma (HCC) cells to apoptosis induced by cisplatin and paclitaxel decreased. However, after DLGAP5 was knockdown, the sensitivity of HCC cells to cisplatin and paclitaxel treatment was conspicuously increased. The results suggested that DLGAP5 was likely a survival protein whose expression level appeared to be critical to the response of tumor cells to chemotherapy drugs  (Kuo et al., 2012; Hewit et al., 2018; Kuo, Lu & Chao, 2011). On the other hand, the high expression of DLGAP5 in prostate cancer enhanced the destabilization of p53 and ATM proteins mediated by DLGAP5, thereby inhibiting gamma radiation-induced apoptosis (Hassan et al., 2016). Although no related studies have performed in endometrial cancer, further exploration for the prognostic value of DLGAP5 may yield far-reaching implications.

**Figure 8:**
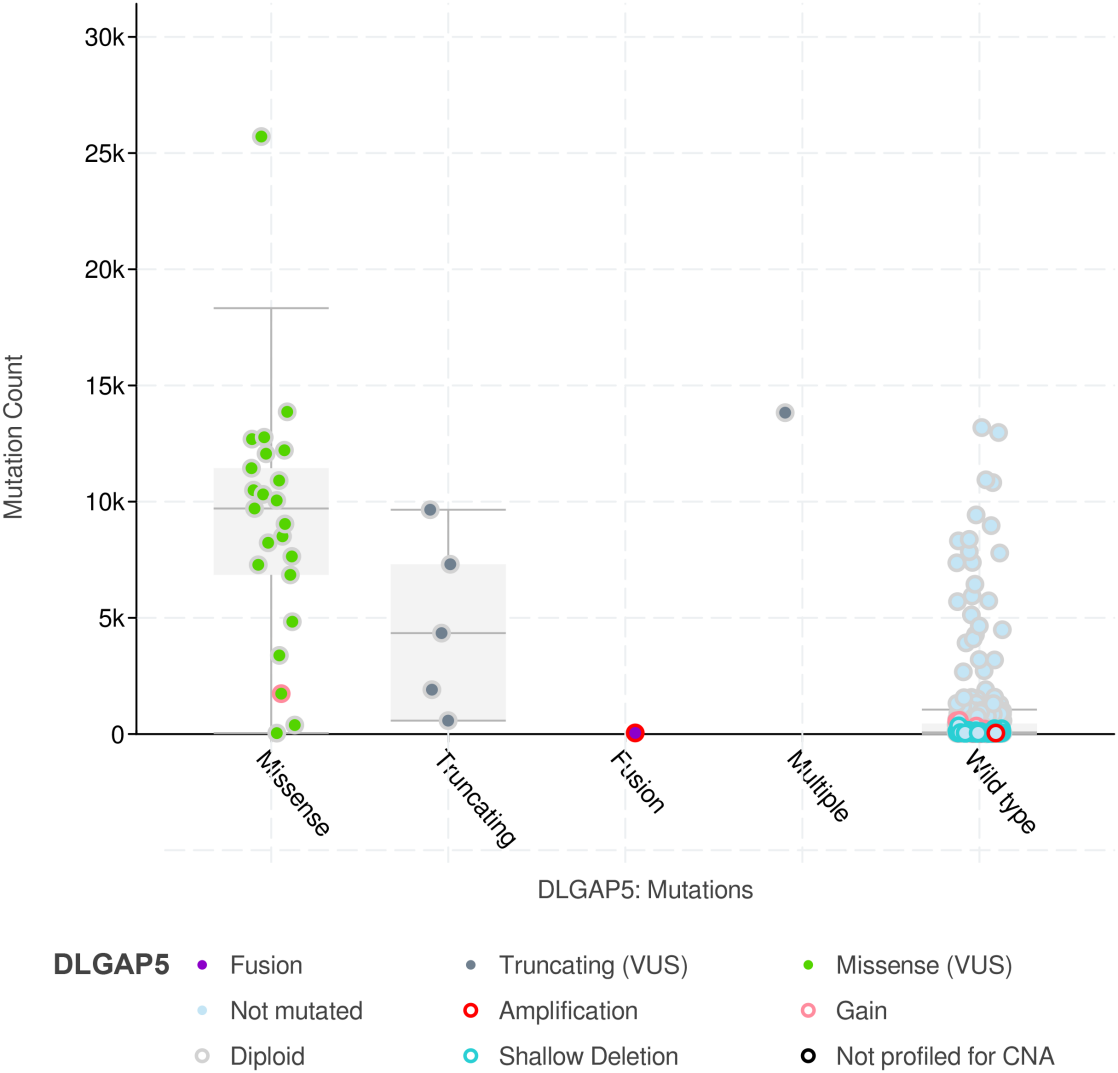
The types and count distribution of somatic mutations in DLGAP5 mutated endometrial cancer. *X* axis shows mutation type of DLGAP5. *Y* axis shows mutation count of clinical attribute.

Besides, there is an interesting phenomenon here. DLGAP5 mutation was found in 6% of TCGA endometrial cancer samples, and the somatic mutation frequency accounted for 5.9%, most of which are missense mutations and relatively dispersed (Fig. 8). While somatic mutations may result in the loss of the original function of the DLGAP5-encoded polypeptide chains, resulting in abnormal function of some of the post-transcriptional proteins. Therefore, we speculate that DLGAP5 may lose its function of maintaining cell cycle and division when missense mutation occurs. Due to the vigorous proliferation requirement of tumor cells, there is a compensatory elevation of DLGAP5 expression. This may be a potential mechanism for DLGAP5 overexpression in endometrial cancer, which needs to be clarified in further studies.

## Conclusions

To sum up, we used TCGA-UCEC and HPA to find that both RNA and protein expression levels of DLGAP5 in endometrial cancer tissues were higher than normal endometrial tissues, and the consistency was verified in clinical samples. At the same time, the results revealed that the expression of DLGAP5 was firmly related to the poor prognosis of patients with endometrial cancer. In depth studies had found that DLGAP5 was an essential and conserved cell cycle regulating gene, whose expression level was affected by the degree of promoter methylation, controlled by hypoxia-control, and could enhance the resistance of tumor cells to chemotherapy and radiotherapy. Based on the findings of the above studies, DLGAP5, as a potential clinical marker and prognostic predictor, has excellent significance for the diagnosis of endometrial cancer and the prediction of the poor OS.

##  Supplemental Information

10.7717/peerj.10433/supp-1 Supplemental Information 1 All the differentially expressed genes in GSE17025 obtained under the conditions set in this study

10.7717/peerj.10433/supp-2 Supplemental Information 2 All the differentially expressed genes in GSE39099 obtained under the conditions set in this study

10.7717/peerj.10433/supp-3 Supplemental Information 3 All the differentially expressed genes in GSE63678 obtained under the conditions set in this study

10.7717/peerj.10433/supp-4 Supplemental Information 4 HTSeq-FPKM levels of DLGAP5 in clinical samples of TCGA-UCEC

10.7717/peerj.10433/supp-5 Supplemental Information 5 Raw data of RT-PCR

10.7717/peerj.10433/supp-6 Supplemental Information 6 These R codes were used by GEO2R to analyze the differential expression of genes in two groups of samples This project categorized eligible data from GSE17025 microarray into normal endometrial and endometrial cancer groups. All of the following codes are downloaded from the GEO2R website and the GEO database owns the copyright to these scripts.

10.7717/peerj.10433/supp-7 Supplemental Information 7 These R codes were used by GEO2R to analyze the differential expression of genes in two groups of samples This project categorized eligible data from GSE39099 microarray into normal endometrial and endometrial cancer groups. All of the following codes are downloaded from the GEO2R website and the GEO database owns the copyright to these scripts.

10.7717/peerj.10433/supp-8 Supplemental Information 8 These R codes were used by GEO2R to analyze the differential expression of genes in two groups of samples This project categorized eligible data from GSE63678 microarray into normal endometrial and endometrial cancer groups. All of the following codes are downloaded from the GEO2R website and the GEO database owns the copyright to these scripts.

10.7717/peerj.10433/supp-9 Supplemental Information 9 TCGA raw data of ROC curve

10.7717/peerj.10433/supp-10 Supplemental Information 10 The ROC curve of DLGAP5

10.7717/peerj.10433/supp-11 Supplemental Information 11 Raw data of Fig. 8

## Additional Information and Declarations

### Data Availability

The following information was supplied regarding data availability: DLGAP5 data are available at TCGA (https://portal.gdc.cancer.gov/genes/ENSG00000126787) and the immunohistochemical and survival analysis data are available from HPA (https://www.proteinatlas.org/ENSG00000126787-DLGAP5) and from the Kaplan-Meier Plotter (http://kmplot.com/analysis/index.php). NCBI GEO microarray datasets (GSE17025, GSE39099, GSE63678) were processed using the online tool GEO2R (http://www.ncbi.nlm.nih.gov/geo/geo2r). The raw measurements and R codes are available in the Supplementary Files. The runtime environment for the R codes is based on the GEO2R online tool (https://www.ncbi.nlm.nih.gov/geo/geo2r/), not locally. Running this code in R will cause the program to report an error because the runtime environment does not match. The R code was made available for download primarily to demonstrate the rationale for the work involving this component.

## References

[ref-1] Barrett T, Wilhite SE, Ledoux P, Evangelista C, Kim IF, Tomashevsky M, Marshall KA, Phillippy KH, Sherman PM, Holko M, Yefanov A, Lee H, Zhang N, Robertson CL, Serova N, Davis S, Soboleva A (2013). NCBI GEO: archive for functional genomics data sets–update. Nucleic Acids Research.

[ref-2] Bray F, Ferlay J, Soerjomataram I, Siegel RL, Torre LA, Jemal A (2018). Global cancer statistics 2018: GLOBOCAN estimates of incidence and mortality worldwide for 36 cancers in 185 countries. CA: a Cancer Journal for Clinicians.

[ref-3] Chen J-MM, Chiu S-C, Wei T-YW, Lin S-Y, Chong C-M, Wu C-C, Huang J-Y, Yang S-T, Ku C-F, Hsia J-Y, Yu C-TR (2015). The involvement of nuclear factor- *κ*appaB in the nuclear targeting and cyclin E1 upregulating activities of hepatoma upregulated protein. Cellular Signalling.

[ref-4] Chen X, Thiaville MM, Chen L, Stoeck A, Xuan J, Gao M, Shih I-M, Wang T-L (2012). Defining NOTCH3 target genes in ovarian cancer. Cancer Research.

[ref-5] Day RS, McDade KK, Chandran UR, Lisovich A, Conrads TP, Hood BL, Kolli VSK, Kirchner D, Litzi T, Maxwell GL (2011). Identifier mapping performance for integrating transcriptomics and proteomics experimental results. BMC Bioinformatics.

[ref-6] Espinoza I, Sakiyama MJ, Ma T, Fair L, Zhou X, Hassan M, Zabaleta J, Gomez CR (2016). Hypoxia on the expression of hepatoma upregulated protein in prostate cancer cells. Frontiers in Oncology.

[ref-7] Gavet O, Pines J (2010). Progressive activation of CyclinB1-Cdk1 coordinates entry to mitosis. Developmental Cell.

[ref-8] Gomez CR, Kosari F, Munz J-M, Schreiber CA, Knutson GJ, Ida CM, El Khattouti A, Karnes RJ, Cheville JC, Vasmatzis G, Vuk-Pavlović S (2013). Prognostic value of discs large homolog 7 transcript levels in prostate cancer. PLOS ONE.

[ref-9] Győrffy B, Lánczky A, Szállási Z (2012). Implementing an online tool for genome-wide validation of survival-associated biomarkers in ovarian-cancer using microarray data from 1287 patients.

[ref-10] Hassan M, El Khattouti A, Ejaeidi A, Ma T, Day WA, Espinoza I, Vijayakumar S, Gomez CR (2016). Elevated expression of hepatoma up-regulated protein inhibits *γ*-irradiation-induced apoptosis of prostate cancer cells. Journal of Cellular Biochemistry.

[ref-11] Hewit K, Sandilands E, Martinez RS, James D, Leung HY, Bryant DM, Shanks E, Markert EK (2018). A functional genomics screen reveals a strong synergistic effect between docetaxel and the mitotic gene DLGAP5 that is mediated by the androgen receptor. Cell Death & Disease.

[ref-12] Koffa MD, Casanova CM, Santarella R, Köcher T, Wilm M, Mattaj IW (2006). HURP is part of a Ran-dependent complex involved in spindle formation. Current Biology.

[ref-13] Kuo T-C, Chang P-Y, Huang S-F, Chou C-K, Chao CC-K (2012). Knockdown of HURP inhibits the proliferation of hepacellular carcinoma cells via downregulation of gankyrin and accumulation of p53. Biochemical Pharmacology.

[ref-14] Kuo T-C, Lu H-P, Chao CC-K (2011). The tyrosine kinase inhibitor sorafenib sensitizes hepatocellular carcinoma cells to taxol by suppressing the HURP protein. Biochemical Pharmacology.

[ref-15] Le NQK, Yapp EKY, Ho Q-T, Nagasundaram N, Ou Y-Y, Yeh H-Y (2019a). iEnhancer-5Step: identifying enhancers using hidden information of DNA sequences via Chou’s 5-step rule and word embedding. Analytical Biochemistry.

[ref-16] Le NQK, Yapp EKY, Nagasundaram N, Chua MCH, Yeh H-Y (2019b). Computational identification of vesicular transport proteins from sequences using deep gated recurrent units architecture. Computational and Structural Biotechnology Journal.

[ref-18] Liao W, Liu W, Yuan Q, Liu X, Ou Y, He S, Yuan S, Qin L, Chen Q, Nong K, Mei M, Huang J (2013). Silencing of DLGAP5 by siRNA significantly inhibits the proliferation and invasion of hepatocellular carcinoma cells. PLOS ONE.

[ref-17] Liao P, Zeng SX, Zhou X, Chen T, Zhou F, Cao B, Jung JH, Del Sal G, Luo S, Lu H (2017). Mutant p53 gains its function via c-Myc activation upon CDK4 phosphorylation at serine 249 and consequent PIN1 binding. Molecular Cell.

[ref-19] Liu J, Meng H, Li S, Shen Y, Wang H, Shan W, Qiu J, Zhang J, Cheng W (2019a). Identification of potential biomarkers in association with progression and prognosis in epithelial ovarian cancer by integrated bioinformatics analysis. Frontiers in Genetics.

[ref-20] Liu J, Zhou S, Li S, Jiang Y, Wan Y, Ma X, Cheng W (2019b). Eleven genes associated with progression and prognosis of endometrial cancer (EC) identified by comprehensive bioinformatics analysis. Cancer Cell International.

[ref-21] Livak KJ, Schmittgen TD (2001). Analysis of relative gene expression data using real-time quantitative PCR and the 2(-Delta Delta C(T)) Method. Methods.

[ref-22] Navarro FJ, Nurse P (2012). A systematic screen reveals new elements acting at the G2/M cell cycle control. Genome Biology.

[ref-23] Pappa KI, Polyzos A, Jacob-Hirsch J, Amariglio N, Vlachos GD, Loutradis D, Anagnou NP (2015). Profiling of discrete gynecological cancers reveals novel transcriptional modules and common features shared by other cancer types and embryonic stem cells. PLOS ONE.

[ref-24] Platani M, Trinkle-Mulcahy L, Porter M, Jeyaprakash AA, Earnshaw WC (2015). Mio depletion links mTOR regulation to Aurora A and Plk1 activation at mitotic centrosomes. Journal of Cell Biology.

[ref-25] Rasmussen AH, Rasmussen HB, Silahtaroglu A (2017). The DLGAP family: neuronal expression, function and role in brain disorders. Molecular Brain.

[ref-26] Schneider MA, Christopoulos P, Muley T, Warth A, Klingmueller U, Thomas M, Herth FJF, Dienemann H, Mueller NS, Theis F, Meister M (2017). AURKA, DLGAP5, TPX2, KIF11 and CKAP5: five specific mitosis-associated genes correlate with poor prognosis for non-small cell lung cancer patients. International Journal of Oncology.

[ref-27] Shi Y-X, Yin J-Y, Shen Y, Zhang W, Zhou H-H, Liu Z-Q (2017). Genome-scale analysis identifies NEK2, DLGAP5 and ECT2 as promising diagnostic and prognostic biomarkers in human lung cancer. Scientific Reports.

[ref-28] Siegel RL, Miller KD, Jemal A (2019). Cancer statistics, 2019. CA: a Cancer Journal for Clinicians.

[ref-29] Strohmaier H, Spruck CH, Kaiser P, Won KA, Sangfelt O, Reed SI (2001). Human F-box protein hCdc4 targets cyclin E for proteolysis and is mutated in a breast cancer cell line. Nature.

[ref-30] Tagal V, Wei S, Zhang W, Brekken RA, Posner BA, Peyton M, Girard L, Hwang T, Wheeler DA, Minna JD, White MA, Gazdar AF, Roth MG (2017). SMARCA4-inactivating mutations increase sensitivity to Aurora kinase A inhibitor VX-680 in non-small cell lung cancers. Nature Communications.

[ref-31] Tsou A-P, Yang C-W, Huang C-YF, Yu RC-T, Lee Y-CG, Chang C-W, Chen B-R, Chung Y-F, Fann M-J, Chi C-W, Chiu J-H, Chou C-K (2003). Identification of a novel cell cycle regulated gene, HURP, overexpressed in human hepatocellular carcinoma. Oncogene.

[ref-32] Uhlen M, Zhang C, Lee S, Sjöstedt E, Fagerberg L, Bidkhori G, Benfeitas R, Arif M, Liu Z, Edfors F, Sanli K, Oksvold P, Feilitzen K von, Lundberg E, Hober S, Nilsson P, Mattsson J, Schwenk JM, Brunnström H, Glimelius B, Sjöblom T, Edqvist P-H, Djureinovic D, Micke P, Lindskog C, Mardinoglu A, Ponten F (2017). A pathology atlas of the human cancer transcriptome. Science.

[ref-33] Wang Q, Chen Y, Feng H, Zhang B, Wang H (2018). Prognostic and predictive value of HURP in non-small cell lung cancer. Oncology Reports.

[ref-34] Wong J, Fang G (2006). HURP controls spindle dynamics to promote proper interkinetochore tension and efficient kinetochore capture. Journal of Cell Biology.

[ref-35] Zhang W, Gao L, Wang C, Wang S, Di Sun, Li X, Liu M, Qi Y, Liu J, Lin B (2020). Combining bioinformatics and experiments to identify and verify key genes with prognostic values in endometrial carcinoma. Journal of Cancer.

