# Supplementary figures and images for: Identification and prognostic value of DLGAP5 in endometrial cancer
Source: PeerJ. 2020 Nov 27;8:e10433. doi: 10.7717/peerj.10433 (PMC7703392; doi:10.7717/peerj.10433)

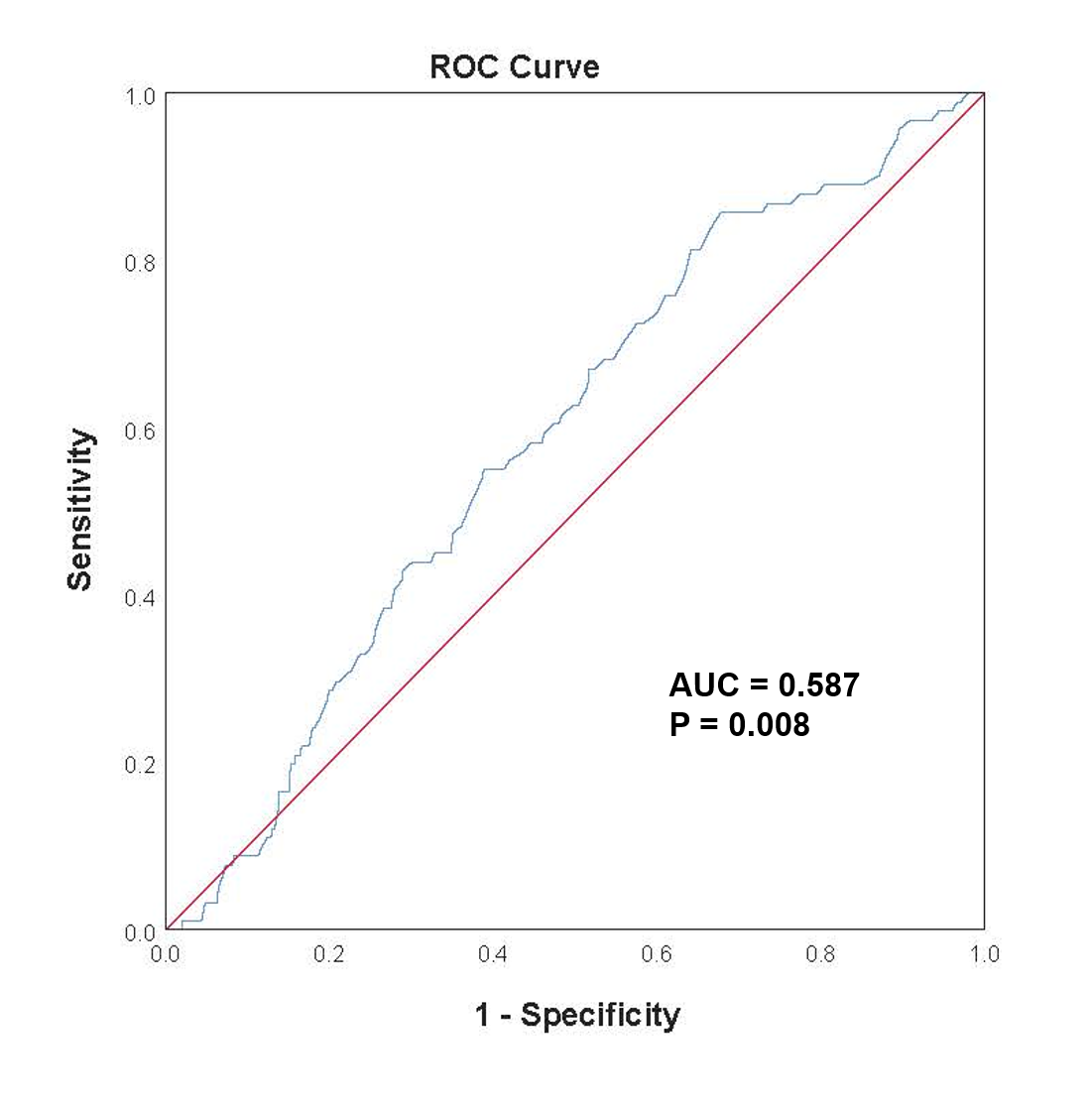

Supplement: Supplemental Information 10 [file peerj-08-10433-s010.png]
